# Tumor-Derived Microvesicles Enhance Cross-Processing Ability of Clinical Grade Dendritic Cells

**DOI:** 10.3389/fimmu.2018.02481

**Published:** 2018-11-05

**Authors:** Marco Dionisi, Claudia De Archangelis, Federico Battisti, Hassan Rahimi Koshkaki, Francesca Belleudi, Ilaria Grazia Zizzari, Ilary Ruscito, Christian Albano, Alessandra Di Filippo, Maria Rosaria Torrisi, Pierluigi Benedetti Panici, Chiara Napoletano, Marianna Nuti, Aurelia Rughetti

**Affiliations:** ^1^Department of Experimental Medicine, “Sapienza” University of Rome, Rome, Italy; ^2^Department of Clinical and Molecular Medicine, Laboratory affiliated to Istituto Pasteur Italia - Fondazione Cenci Bolognetti, “Sapienza” University of Rome, Rome, Italy; ^3^European Competence Center for Ovarian Cancer, Department of Gynecology, Campus Virchow Klinikum, Charité - Universitätsmedizin Berlin, Corporate Member of Freie Universität Berlin, Humboldt-Universität zu Berlin, Berlin Institute of Health, Berlin, Germany; ^4^U.O.C. Genetica medica e Diagnostica cellulare avanzata, S. Andrea University Hospital, Rome, Italy; ^5^Department of Gynecology-Obstetrics and Urology, “Sapienza” University of Rome, Rome, Italy

**Keywords:** dendritic cells, DC vaccine, microvesicles, cancer immunotherapy, antigen processing, phagosome, tumor antigens, MUC1

## Abstract

Tumor cells release extracellular microvesicles (MVs) in the microenvironment to deliver biological signals to neighboring cells as well as to cells in distant tissues. Tumor-derived MVs appear to play contradictory role promoting both immunosuppression and tumor growth and both evoking tumor specific immune response. Recent evidences indicate that tumor-derived MVs can positively impact Dendritic Cells (DCs) immunogenicity by reprogramming DC antigen processing machinery and intracellular signaling pathways, thus promoting anti-tumor response. DCs are considered pivot cells of the immune system due to their exclusive ability to coordinate the innate and acquired immune responses, cross-present exogenous antigens, and prime naïve T cells. DCs are required for the induction and maintenance of long-lasting anti-tumor immunity and their exploitation has been extensively investigated for the design of anti-tumor vaccines. However, the clinical grade culture conditions that are required to generate DCs for therapeutic use can strongly affect their functions. Here, we investigated the immunomodulatory impact of MVs carrying the MUC1 tumor glycoantigen (MVs_MUC1_) as immunogen formulation on clinical grade DCs grown in X-VIVO 15 (X-DCs). Results indicated that X-DCs displayed reduced performance of the antigen processing machinery in term of diminished phagocytosis and acidification of the phagosomal compartment suggesting an altered immunogenicity of clinical grade DCs. Pulsing DCs with MVs_MUC1_ restored phagosomal alkalinization, triggering ROS increase. This was not observed when a soluble MUC1 protein was employed (rMUC1). Concurrently, MVs_MUC1_ internalization by X-DCs allowed MUC1 cross-processing. Most importantly, MVs_MUC1_ pulsed DCs activated IFNγ response mediated by MUC1 specific CD8^+^ T cells. These results strongly support the employment of tumor-derived MVs as immunogen platforms for the implementation of DC-based vaccines.

## Introduction

Dendritic Cells (DCs) are antigen presenting cells (APCs) crucial for the promotion and maintenance of the anti-tumor immune response due to their ability to coordinate innate and adaptive immune response and to activate T cells inducing immune memory (1, 2). DCs are equipped with a variety of receptors able to sense tissue and cellular damage; they are endowed with an unique and powerful antigen processing machinery that enable them to crossprocess and present antigens; lastly, they display a complex pattern of costimulatory/inhibitory receptors/ligands that regulate interactions with effector immune cells (3). These biological features empower DCs to perform T cell cross priming thus activating both CD4^+^ and CD8^+^ T cells (4, 5).

Indeed, the exploitation of DCs in order to activate, redirect and boost the immune response against the tumor is one of the first strategies foreseen for anti-cancer immunotherapeutic purposes (6–8). Among the different biological and experimental parameters that have to be considered in the design of DC-based vaccines, antigen selection and modality of antigen loading are key points that still require to be improved to obtain an optimal DC vaccine (9). Optimization of immunogen formulation is also crucial to compensate those biological changes that characterized DCs grown in clinical grade culture conditions and that could affect the overall immunostimulatory ability of DCs (10, 11).

Recently, cell-derived extracellular microvesicles (MVs) have been regarded as an interesting option for the formulation of DC-based vaccines.

Release of MVs is an inter-cellular communication modality that allows the delivery of molecular signals into the microenvironment triggering metabolic reprogramming of the acceptor cells even in distant tissue districts, overcoming cell-to-cell contact (12, 13). Distinct MV subsets are shed by each cell and are heterogeneous for biogenesis, size, and molecular cargo components (14).

During tumor transformation, MVs released by the transforming cells exert apparently contradictory effects on host immune response. Tumor MVs have been show to promote tumor growth, modulate matrix components and trigger immunosuppression thus leading to invasion and metastasis (15–17). On the other hand, it is clear that tumor MVs can activate and promote long lasting anti-tumor immune responses (18–20). In mouse models tumor-derived MVs have been shown to be optimal immunogens for immunotherapeutic vaccination both in prophylactic and therapeutic settings (21). In addition, the immunogenity of tumor MVs appeared to be superior to the one of soluble antigens since they trigger a more efficient anti-tumor immune response than soluble antigens (22). Recent evidences suggest that immunogenicity of tumor-derived MVs observed *in vivo* may be also dependent by the antigenic and molecular signals that tumor MVs convey to DCs. Tumor-derived MVs are source of tumor antigen repertoire and have been shown to reprogram DC antigen processing and signaling pathways, resulting in increased DC immunogenicity (23–26).

In this work, we investigated whether MV based immune formulations could restore the biological performance of DCs differentiated in X-VIVO 15 serum free medium (X-DCs). Results indicated that X-DCs displayed a reduced performance of the antigen processing machinery as compared to standard DCs (S-DCs) i.e. reduced phagocytosis and acidification of the phagosomal compartment.

The antigen processing ability of both X-DCs and S-DCs was evaluated employing two distinct formulations of the MUC1 tumor glycoantigen: a soluble recombinant MUC1 glycoprotein (rMUC1) and tumor-derived MVs carrying MUC1 (MVs_MUC1_), isolated from the MUC1 transfected DG75 cell line (27). Results indicated that only MVs_MUC1_ up-take restored the phagosomal alkalinization of X-DCs and this event was dependent by the modulation of the phagosomal radical oxigen species. Moreover, MUC1 cross-processing to HLA class I compartment was still occurring in X-DCs upon MV pulsing and IFNγ response mediated by MUC1 specific CD8^+^T cells could be triggered by MVs_MUC1_ pulsed DCs. These results strongly suggest that the employment of MVs as immunogens for DC-based vaccine may contribute to restore the functionality of antigen processing machinery in clinical grade DCs, besides transferring the entire antigenic *repertoire* of tumor cells. Also, these evidences support further exploitation of MVs based formulation as off the shelf/cell free-immunogens for the implementation of DC-based vaccines.

## Materials and methods

### Recombinant MUC1 glycoprotein (rMUC1)

rMUC1 was produced by CHO-K1 cells (ATCC CRL-9618) transfected with a MUC1-murine-IgG2a fusion cDNA construct containing 16 MUC1 tandem repeats. The secreted MUC1-IgG was highly sialylated due to the translational modifications occurring in CHO-K1 cells. The rMUC1 glycoprotein was purified from cell culture supernatant by anion exchange chromatography after cleavage of the Fc portion by enterokinase treatment (28).

### Dendritic cell generation

Dendritic cells were generated as previously described (29). Briefly, Peripheral Blood Mononuclear Cells (PBMCs) were isolated from buffy coat of healthy donors, by Ficoll-Hypaque gradient (Lympholite-H, Canada) (Policlinico Umberto I Ethics Committee- Protocol nr. 4214/2016; written informed consent was obtained from the subjects in accordance with Declaration of Helsinki). CD14^+^ monocytes were isolated from PBMCs by immunoselection kit (StemCell Technologies Inc., CA, USA) and cultured with RPMI 1640 (Sigma-Aldrich, MO, USA) complemented with 10% Fetal Bovine Serum (FBS; Euroclone, Italy) (S-DCs) or in clinical grade X-VIVO 15 culture medium (X-DCs) (Lonza, Switzerland) in the presence of 500 UI/mL of GM-CSF and 2,000 UI/mL of IL-4 (R&D Systems, USA) (day 0 and 2). Immature DCs (iDCs) grown in X-VIVO 15 were indicated as X-DCs, while iDCs grown in the presence of FBS were indicated as S-DCs. Cells were maintained in a humidified atmosphere at 37°C and 5% CO_2_ (HERAcell 150, AHSI, Italy). At day 5, iDCs were matured (mDCs) by adding rhIL-1β (1,000 UI/mL−10 ng/mL), IL-6 (1,000 UI/mL−10 ng/mL), TNF-α (465 UI/mL−10 ng/mL) and prostaglandin E_2_ (1 μg/mL) (all from R&D Systems, USA) for 16 h.

mDCs grown in the presence of RPMI + 10% FBS or X-VIVO 15 were employed only for CD8^+^T cells activation and ELISpot assay. Immature X-DCs and S-DCs were employed for all the other experiments.

### Cell lines

DG75 cell line and MUC1-DG75–transfected cells were cultured as previously described in RPMI + 10% FBS (Euroclone) without or with neomycin (1 mg/mL; Invitrogen, CA, USA), respectively (27). Before MVs production, MUC1-DG75 cells were analyzed for the expression of MUC1 by flow cytometry (see below).

### Flow cytometry

DC phenotype staining was performed using the following antibodies directly conjugated with fluorescein isothiocyanate (FITC) or phycoerythrin (PE): IgG_1_-FITC and IgG_1_-PE as isotype controls (both from Biolegend); anti-HLAII-DR-FITC, anti-CD86-FITC, anti-CD83-PE (all from BD Biosciences), anti-CD40-PE, anti-CD14-PE, and anti-CCR7-FITC (all from Biolegend). DCs (2 × 10^5^ cells/50μL sample) were incubated with conjugated MoAb (according to the manufacturer's recommendation) for 30 min at room temperature (RT). After washing (in 2 mL of PBS w/o Mg^++^ and Ca^++^, centrifuged at 250 × g for 5 min), cell pellet was resuspended in PBS (100 μL); at least 1 × 10^4^ events were evaluated using a FACSCanto II flow cytometer running FACSDiva data acquisition and analysis software (Becton Dickinson).

To evaluate MUC1 expressed by MUC1-DG75 cells, 1 × 10^5^ cells were incubated with MoAb Ma552 (1:40; Monosan, Netherlands, 50 μL/sample) for 30 min at RT and binding revealed with FITC-conjugated anti-mouse antibody (1:600; Jackson-Immunoresearch Laboratories, PA, USA). MoAb MOPC21 (1:100; Sigma-Aldrich, 50 μL/sample) was employed as isotype control.

### MV purification

MVs were purified from cell culture supernatant of MUC1-DG75 (MVs_MUC1_) or DG75 cells (MVs_DG75_) (23). To generate MVs, cells were cultured 3.5 × 10^5^ cells/mL in RPMI 1640 (Sigma-Aldrich) complemented with 2% FBS (Euroclone) for 48 h. Supernatant (70 mL/tube) underwent to serial centrifugation steps at 4°C (250 × g for 10 min, 550 × g for 30 min, 1,500 × g for 30 min) (Allegra™ 6R Centrifuges, Beckman Coulter, USA). Then supernatant was ultracentrifuged at 10,000 × g for 30 min at 4°C. Following transfer in fresh tube, the supernatant was ultracentrifuged at 100,000 × g for 1 h at 4°C (Type 35 rotor, Beckman Coulter, USA). Following the last ultracentrifugation step, supernatant was discarded and the final pellet containing MVs was gently resuspended in PBS w/o Mg^++^ and Ca^++^ (100 μL/pellet), aliquoted and stored at −20°C. Protein concentration was measured by Bradford assay (Bio-Rad Laboratories, USA). An average of 0. 95 μg/μL of MVs_MUC1_ and 0.91 μg/μL of MVs_DG75_ was obtained.

### MV characterization

Size determination of MVs_MUC1_ was performed by Nanoparticle Tracking Analysis (NTA) technology (30). MVs were thawed on ice and diluted in PBS between 1:500 and 1:20,000 to achieve the optimal number of MVs/mL. Three videos (30 s each) were recorded for each sample loading, employing the NanoSight NS300 instrument (Malvern Instruments Ltd, Malvern, UK). Measurements were performed employing the NTA 2.3 analytical software. Results were shown as the average of the three recordings.

MUC1 expression on MVs_MUC1_ was evaluated by flow cytometry. MVs_MUC1_ (5 μg/sample) were incubated with the anti-MUC1 MoAb Ma552 (Monosan) (1:100 for 30 min, 50 μL/sample, RT). After washing in PBS w/o Mg^++^ and Ca^++^(1 mL/sample, 30 min at 13,000 rpm, RT), MVs_MUC1_ were incubated with FITC-conjugated anti-mouse antibody (1:600; Jackson-Immunoresearch Laboratories, 50 μL/sample). MoAb MOPC21 (1:100; Sigma-Aldrich) was employed as isotype control. To exclude background noise, flow cytometry analysis was performed setting the lowest Forward Scatter Threshold [300] and the highest FSC/SSC voltage. A total of 30,000 events were acquired with low flow rate, using a FACSCanto II flow cytometer running FACSDiva data acquisition and analysis software (Becton Dickinson).

### Western blot

MVs_DG75_, MVs_MUC1_ and extract of DG75-MUC1 cell line (obtained by freeze and thaw method) (30 μg for sample) were separated on 4–12% SDS-PAGE (95V, 220 mA for 90 min at RT) and blotted onto nitrocellulose transfer membrane (Schleicher und Schuell, DE). Prestained protein ladder (10 μL) by Nippon Genetics Europe GmbH was used. After blocking (5% BSA in PBS), membranes were incubated with anti-MUC1 MoAb Ma552 (1:100, 1 h at RT; Monosan), followed by anti-mouse Fc peroxidase-conjugated antibody (1:20,000; 1 h at RT;Jackson ImmunoResearch, USA). Protein bands were detected with enhanced chemiluminescence reagents (ECL Western Blotting Detection; Amersham Biosciences, UK).

### Measurement of DC phagosomal pH

DC phagosomal pH was measured as previously described (23). Briefly, immature DCs were pulsed (10^6^ cells/100 μL) for 30 min at 37°C in CO_2_-indipendent medium (Gibco-Life Technologies, UK) with 3 μm microbeads (Polysciences Inc., USA) coupled with FITC (1 mg/mL) (pH sensitive, Sigma-Aldrich) and FluoProbes 647 (1 mg/mL) (pH insensitive, Interchim, France). After extensive washing in cold PBS w/o Mg^++^ and Ca^++^to remove not internalized microbeads, cells were incubated at 37°C (“chase”) at different time points (10, 20, 30, 60, and 120 min) in CO_2_-indipendent medium and immediately analyzed by flow cytometry (FACSCanto II, FACSDiva software, Becton Dickinson). A FL1(FITC)/FL4(FluoProbes 647) gate selective for cells that had phagocytosed only one microbead was employed. Values of the ratio between the Mean Fluorescence Intensity (MFI) of FL1(FITC)/FL4(FluoProbes 647) were compared with a standard curve obtained by suspending DCs that had phagocytosed beads, in CO_2_-independent medium at a fixed pH (ranging from pH 5.5 to pH 8) containing 0.1% Triton X-100 (Bio-Rad Laboratories, Inc., Italy).

The effect of MUC1 based immunogens on phagosomal pH of X-DCs was analyzed by pulsing the immature X-DC samples (10^6^ cells/100 μL) for 30 min at 37°C in CO_2_-indipendent medium (Gibco-Life Technologies) with rMUC1 glycoprotein (20 μg/mL) and MVs_MUC1_ (500 μg/mL). Then, the DCs samples were processed as above described. To block NADPH oxidase 2 (NOX2) activity, 10 μM Diphenyleneiodonium chloride (DPI, Sigma-Aldrich) was added to DCs 30 min before MVs pulsing and it was maintained throughout the experiment in each solution the DCs were suspended in.

### Phagocytosis assay

To evaluate phagocytosis capability, DCs (10^6^ cells/100 μL) were pulsed with 3 μm microbeads (Polysciences Inc., USA) coupled with FluoProbes 647 (ROS insensitive, Interchim) for 30 min at 37°C in the growth medium. The samples were then extensively washed in cold PBS to remove not internalized microbeads. The cells were resuspended in growth medium (10^6^ cells/100 μL) and kept at 37°C for 1 h. After washing in cold PBS, samples were analyzed (at least 2 × 10^5^ events) by flow cytometry employing FACScanto II (Becton Dickinson). As control, cells were also kept at 4°C on wet ice to block phagocytosis capability. Phagocytosis was indicated as the percentage of fluorescence positive cells subtracted of the fluorescence signal associated to the corresponding control sample.

### Immunofluorescence microscopy

iDCs (both S-DCs and X-DCs) (10^6^ cells/100 μL) were incubated with rMUC1 glycoprotein (20 μg) or MVs_MUC1_ (500 μg/mL) in growth medium for 2 h or 12 h at 37°C, 5% CO_2_. At the end of incubation, iDCs were washed twice in PBS and were cytospun (8 × 10^4^ cells/sample) and fixed with cold acetone/methanol (1:1; Carlo Erba Reagents, Italy). iDCs were incubated in humid chamber with the anti-MUC1 MoAb Ma552 (1:20, Monosan) for 45 min at RT, washed in PBS (5 min in orbital shaker, 3 times), followed by FITC-conjugated goat anti-mouse F(ab)_2_ for 30 min at RT (1:100). Both dilutions were performed in PBS. MUC1 positive cells were counted (30 fields) for each experimental condition and percentage was expressed as ratio between positive and total cell in the field. Three independent experiments were evaluated.

To study MUC1 cross-processing, the iDCs (both S-DCs and X-DCs) (10^6^ cells/100 μL) were incubated with rMUC1 or MVs_MUC1_ for 12 h as above described. iDCs were then washed and stained for MUC1 expression as above. After PBS rinse (3 times, 5 min, orbital shaker), block of aspecific sites was performed by 15 min incubation with Superblock reagent (50 μL sample/slide). Following removal of the blocking solution, the iDCs were then incubated with MoAbs anti-HLAII-DR (L243 clone, 100 μL of neat supernatant) or rabbit polyclonal antibody anti-calreticulin (1:50; Stressgene, USA) (45 min, RT in the dark) to visualized HLA class II and I compartments, respectively. After washing (PBS, 3 times, 5 min, orbital shaker), samples were then incubated with Texas red–conjugated goat anti-mouse or anti-rabbit antibody, respectively (1:200, 30 min in the dark; Jackson ImmunoResearch, USA).

Fluorescence signals were visualized with an Axiovert 200 inverted microscope (Zeiss, Germany); cells were scanned in a series of 0.5 μm sequential sections with an ApoTome System (Zeiss) and images were all acquired by the digital camera Axio CAM MRm (Zeiss). Image analysis was performed by the Axiovision software (Zeiss) and a reconstruction of a selection of three central optical sections was shown in each figure. Quantitative analysis of the extent of colocalization of fluorescence signals was performed using the Axiovision software (Zeiss). The mean ± SE percent of colocalization was calculated analyzing a minimum of 30 cells for each treatment randomly taken from three independent experiments.

### MUC1^+^ CD8^+^ T cell enrichment and IFNγ ELISpot

MUC1^+^ CD8^+^ T cell enrichment and IFNγ ELISpot were performed as previously described (23). Briefly, PBMCs of a MUC1 vaccinated ovarian cancer patient (open-label phase I/II safety clinical peptide vaccination trial (31), approved by Policlinico Umberto I Ethics Committee and Italian National Institute of Health/protocol no. LITRM/DIMIGE05/01; Ethical Committee Protocol nr. 1454/2008) were isolated by Ficoll/Hypaque density gradient. Written informed consent was obtained from the subjects in accordance with Declaration of Helsinki. CD8^+^ T cells were purified by CD8^+^ positive immunoselection kit (Stemcell Technologies, USA) and kept in RPMI + 5% FBS at 37°, 5% CO_2_. The CD8^−^ cell fraction (4 × 10^6^ cell/mL) was incubated overnight (o/n) with 50 μg/mL of MUC1_159−167_ peptide (SAPDNRPAL) (ClinAlfa, Switzerland) and 5 μg/mL β2-microglobulin (Sigma Aldrich) in RPMI + 1% FBS, at 37°, 5% CO_2_. The MUC1_159−167_ peptide specifically binds HLAI-A2 groove (31). The following day, CD8^−^ cells were irradiated (30 Gy) and plated with autologous CD8^+^ T cells (1:1; 2 × 10^6^ total cells/mL) in RPMI + 5% FBS, supplemented with IL-2 (50 UI/mL, Peprotech, USA) and IL-7 (1,000 UI/mL; R&D System).

After 7 day of co-culture, freshly isolated and MUC1-pulsed autologous PBMCs (generated as above described) were irradiated and added to the culture (1:1), with IL-2 (50 UI/mL, Peprotech, USA) and IL-7 (1,000 UI/mL; R&D System). At the same time, autologous CD14^+^ cells were immunoselected (Stemcell Technologies, USA) and cultured in RPMI + 10% FBS or X-VIVO 15 in the presence of GM-CSF (500 UI/mL) and IL-4 (2,000 UI/mL) (day 0 and 2). At day 5, iDCs (1 × 10^5^ cells/100 μL) were pulsed o/n with MVs_MUC1_ (500 μg/mL), MVs_DG75_ (500 μg/mL) or [MUC1_159−167_ peptide with β2-microglobulin] (50 and 5 μg/mL, respectively). After 2 h pulsing, the DC samples were matured with cytokine cocktail, o/n. Following maturation, mDCs were washed in PBS and added to MUC1^+^ enriched CD8^+^ T cells (1:5, respectively), previously expanded in culture and purified by immunoselection to remove cell debris. Pulsed mDCs/T cells were plated (1 × 10^5^ T cells/2 × 10^4^ DCs/200 μL /well) in duplicate onto the anti-IFNγ-precoated (1:200; BD Biosciences) ELISpot plate (MultiScreen, Merck, Germany), o/n. Unpulsed DCs + T cells were also plated at the same concentration. IFNγ cytokine release was detected with biotinylated anti-IFNγ antibody (1:250, 2 h; BD Biosciences), revealed with streptavidin-alkaline phosphatase (BD Biosciences) (1:1,000, 100 μL /well, 1 h) and chromogen substrate (SIGMA FAST BCIP/NBT, Sigma). Spots were counted using the ImmunoSpot Image Analyzer (Aelvis, Germany).

The average values of the experimental conditions [(DCs + MUC1_159−167_) + CD8^+^T cells] and [(DCs + MVs_MUC1_) + CD8^+^T cells] were subtracted of the average values of the background samples [unpulsed DCs + CD8^+^T cells] and [(DC + MVs_DG75_) + CD8^+^T cells], respectively.

### Statistical analysis

Statistics was performed using GraphPad Prism software, version 6 (GraphPad Software, Inc., USA). Results were expressed as mean values ± SD. *p-*values were calculated using Student's *t-*test when comparing two groups of continuous variables. Significance level was defined as *p*-value <0.05 (^*^p < 0.05; ^**^p < 0.01; ^***^p < 0.005).

## Results

### Dendritic cells for clinical use display a less efficient antigen processing phagosomal machinery

**Figure 1:**
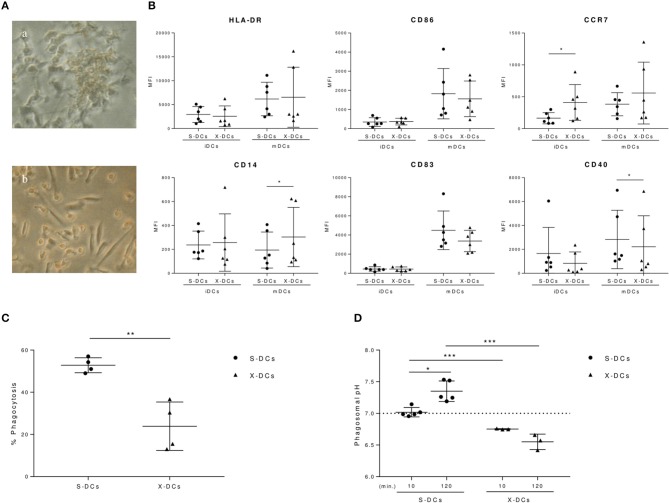
Clinical grade DCs display different biological features compared to standard S-DCs**. (A)** Morphological differences of DCs grown in RPMI + 10%FBS (S-DCs) and in X-VIVO 15 (X-DCs) (a,b, respectively) visualized by phase contrast inverted microscope (ZEISS West Germany IM35, 3,2X). **(B)** Flow cytometry analysis of immature and mature DCs (iDCs and mDCs, respectively) grown in FBS-RPMI or X-VIVO 15 after 6 day culture in the presence of GM-CSF, IL-4. iDCs were matured with IL-6, IL-1β, PGE-2, and TNF-α on day 5. IgG_1_ PE and FITC were used as isotype controls and employed to evaluate fluorescence signal background and set the gate. Results were shown as value of Mean Fluorescence Intensity (MFI) of each phenotypic marker subtracted of the corresponded negative control MFI value and depicted as scatter plot (black circle for S-DCs; black triangles for X-DCs). Statistically significant differences between S-DCs vs X-DCs were indicated (*p < 0.05). (**C)** Phagocytosis of S-DCs and X-DCs was evaluated at 1 h from the internalization of 3 μm FITC/FluoProbes 647 coupled beads by flow cytometry (FACSCanto II, FACSDiva software, BD Biosciences). Results were plotted as percentage of positive cells of experimental samples subtracted of the percentage of positive cells of corresponding cell samples kept at 4°C for 1 h (black circle for S-DCs; black triangles for X-DCs). **(D)** Phagosomal pH of clinical grade X-DCs (three donor, black triangles), is compared to the S-DCs (five donors, black circles) at 10 min and 120 min of chase. Average of the results of each experiment is plotted as black line. Dashed line indicates the pH neutrality value (pH 7). Significance between samples was evaluated by Student's *t* test. (*p < 0.05; **p < 0.01, *** *p* < 0.005).

Serum-free culture conditions employed for generating DCs for anti-tumor vaccination can alter DC phenotype, modifying to some extent their immunogenicity (8, 10, 32). Indeed, DCs grown in the serum free X-VIVO 15 medium (X-DCs) acquired a spindle-like morphology, quite distinct from the one observed in DCs grown in RPMI in the presence of FBS (S-DCs) (Figures 1Ab,a respectively). Results from the phenotypic analysis performed by flow cytometry, showed that at the immature stage, X-DCs expressed significant higher levels of the maturative marker CCR7 chemokine receptor (*p* < 0.05) (Figure 1B). CD14 expression was slightly higher although not significant in immature X-DCs, while no significant change in the expression of other markers was observed between the two DC cultures.

Following maturation, in both DC cultures the activation markers were upregulated, although with a different intensity. Mature X-DCs displayed a reduced expression of CD40 costimulatory molecule (*p* < 0.05) as compared to mature S-DCs, as well as a trend in the reduction of CD86 and CD83 costimulatory molecules could be observed. These changes were accompanied by the significant increase of CD14 in mature X-DCs vs. mature S-DCs (*p* < 0.05). Again a trend in a more pronounced expression of CCR7 marker was still maintained in mature X-DCs (Figure 1B).

Phagocytosis is a crucial biological function of immature DCs and it is a key step for the antigen loading of DCs for cancer vaccines. Phagocytic activity of both immature S-DCs and X-DCs was evaluated by flow cytometry, following the uptake of 3 μm microbeads, conjugated with FluoProbes 647, fluorochrome not affected by changes in pH. After 1 h incubation at 37°C, phagocytosis of X-DCs was significantly reduced as compared to S-DCs (*p* < 0.01) (Figure 1C). Phagosomal activity in DCs is specifically dependent on a mild alkalinization, differently from what is observed in other antigen presenting cells (APCs) such as macrophages.

Kinetic of phagosomal pH in both immature X-DCs and S-DCs was followed by flow cytometry. As shown in Figure 1D, S-DCs presented a neutral phagosomal pH (7.01 pH) that significantly increased after 2 h chase (7.35 pH) (*p* < 0.05).

X-DCs differently behaved: phagosomal pH of X-DCs was significantly lower than S-DCs both at 10 min and 120 min chase (*p* < 0.001). Furthermore, in X-DCs phagosomal pH appeared to decrease during the chase, although not significantly.

These results suggest that clinical grade DCs have a phenotype and a biological behavior that appears to remain at a more immature stage with a more acid phagosomal compartment as compared to standard S-DCs. This feature could contribute to reduce antigen cross-processing efficiency of clinical grade DCs.

### DC uptake of the tumor associated MUC1 antigen carried by MVs increases antigen internalization and induces phagosomal alkalinization

We have recently shown that MV up-take by DCs allows cross-presentation of the MUC1 tumor glycoantigen by triggering a faster alkalinization of DC phagosomal compartment (23). We therefore evaluated whether MV uptake could similarly impact phagosomal pH in the clinical grade X-DCs.

**Figure 2:**
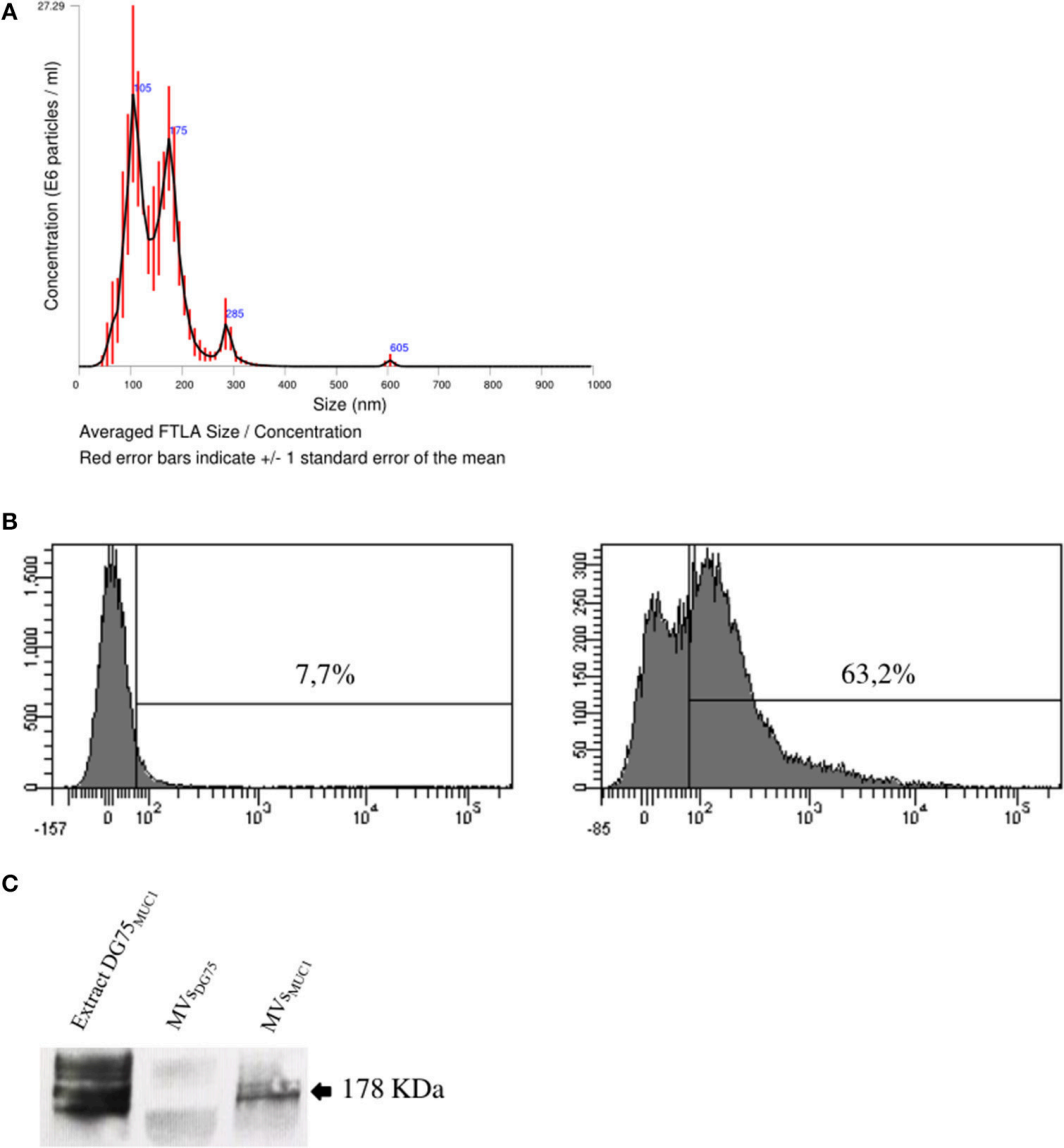
Characterization of tumor-derived microvesicles (MVs_MUC1_). **(A)** Size measurement of MVs shed by the MUC1-transfected DG75 lymphoblastoid cell line (MVs_MUC1_) using Nanosight NS300 that employs Nanoparticles Tracking Analysis (NTA) technology. Results are plotted as graph; *y-axis*: concentration of particles; *x-axis*: size of particles in nanometer. The black curve is obtained by the merge of three independent measurements for each MV sample. **(B)** MUC1 expression in MVs_MUC1_ by flow cytometry. MoAb MOPC21 was employed as isotype control (left). MUC1 expression was detected employing the MoAb Ma552 (right). **(C)** Western Blot analysis to detect MUC1 in MVs_DG75_, MVs_MUC1_ and MUC1-DG75 cell extracts (30 μg/sample) employing the MoAb Ma552. The extract of MUC1-DG75 cell line was used as positive control.

MVs were isolated from the supernatant of MUC1-DG75 cell line (MVs_MUC1_). MV size characterization by Nanoparticles Tracking Analysis (NTA) indicated that MVs_MUC1_ were heterogeneous for size: 3 main vesicle populations could be identified with a size corresponding to 105, 175, and 285 nm (Figure 2A). The MUC1 tumor glycoantigen was a molecular cargo component of the MVs_MUC1_ as characterized by flow cytometry (Figure 2B) and Western blot analysis confirmed the presence of the MUC1 antigen (Figure 2C). A soluble recombinant form of MUC1 glycoprotein (rMUC1) was also employed. The rMUC1 had a glycosylation profile (high level of sialylation), similar to the MUC1 carried by MVs_MUC1_ as defined by pattern reactivity of MoAbs specific for distinct MUC1 glycoforms (27).

**Figure 3:**
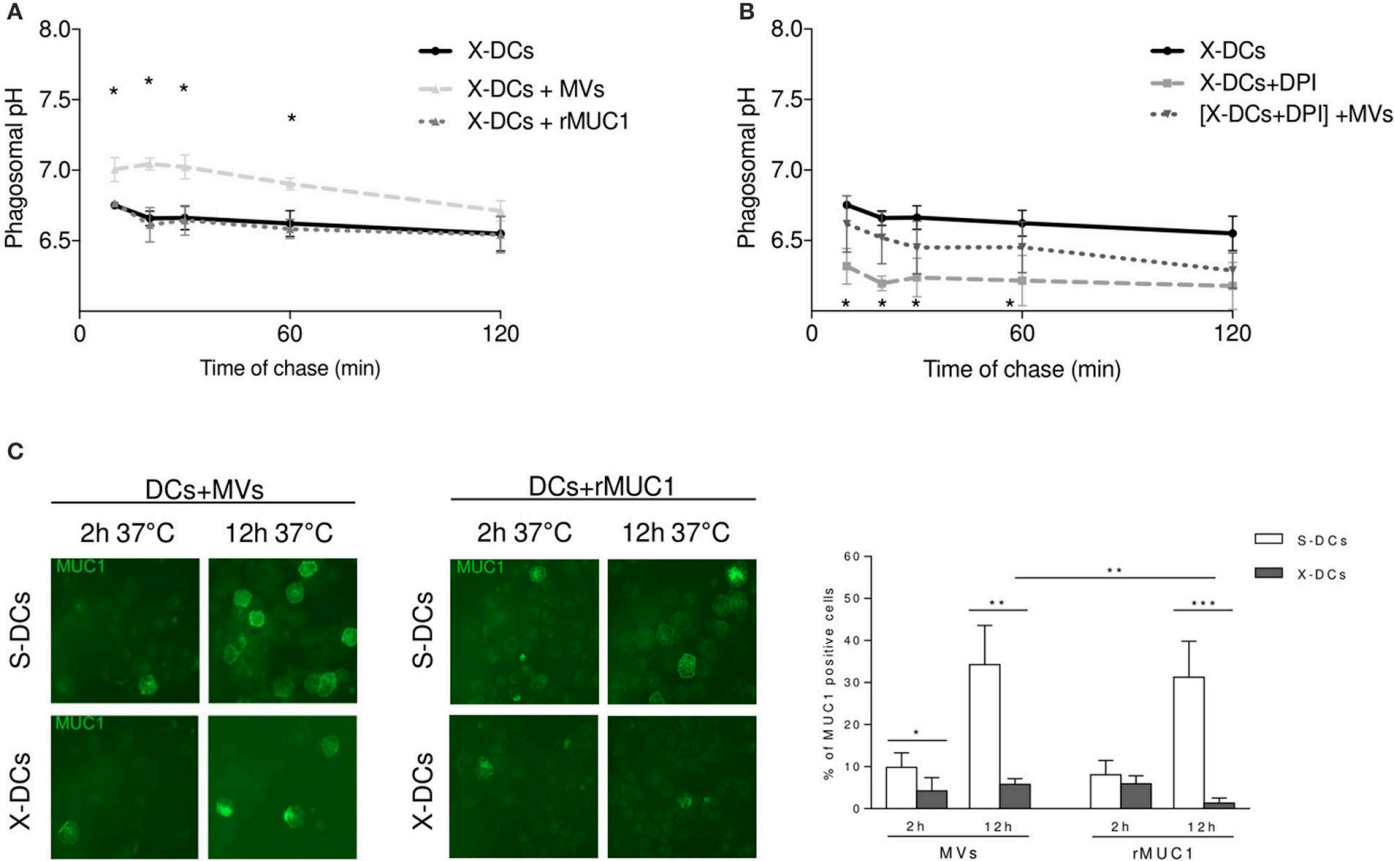
Tumor-derived MVs_MUC1_ efficiently increase X-DC phagosomal pH and transfer MUC1 antigen to X-DCs. **(A)** Kinetic of phagosomal pH (10–120 min chase) of X-DCs, pulsed with soluble rMUC1 or with MVs_MUC1_. Following MVs_MUC1_ uptake, phagosomal pH of X-DCs (light gray dashed line) was significantly increased in the first 60 min of chase (*p* < 0.05) as compared to unpulsed X-DCs (black continuous line). Soluble rMUC1 protein uptake did not alter phagosomal pH of X-DCs (dark gray dashed line). The average ± SD of three independent experiments (3 different donors) was shown. *p < 0.05. **(B)** Phagosomal pH measurement in X-DCs in the presence of 10 μM Diphenyleneiodonium chloride (DPI), NOX2 inhibitor, without or with MVs_MUC1_. DPI treatment decreased pH compared to untreated X-DCs (light gray dashed line vs. black continuous line, respectively). In [DPI-treated X-DCs + MVs_MUC1_] (black dotted line) phagosomal pH was partially restored. The difference between [DPI treated X-DCs] and [DPI-treated X-DCs + MVs_MUC1_] was significant for the first 60 min of chase (*p* < 0.05). Values are mean ± SD of three independent experiments. *p < 0.05; **p < 0.01, ***p < 0.005. **(C)** MUC1 expression in DCs following pulsing with rMUC1 glycoprotein or MVs_MUC1_. S-DCs (first row) and X-DCs grown in X-VIVO 15 (second row) were visualized by immunofluorescence staining after 2 and 12 h incubation, employing the anti-MUC1 MoAb Ma552 (green). The average ± SD of percentage of positive cells (evaluated by counting 30 fields for each experimental condition, three independent experiments) was shown as histograms (White: S-DCs; gray: X-DCs). Uptake by X-DCs was significantly decreased as compared to S-DCs. Within the X-DCs, MVs_MUC1_ uptake was higher than the soluble rMUC1 (*p* < 0.01). *p < 0.05; **p < 0.01, ***p < 0.005.

To evaluate whether phagosomal compartment was differentially modulated by the up-take of the two distinct MUC1 immunogens, X-DCs were pulsed with MVs_MUC1_ and the soluble rMUC1 glycoprotein for 30 min (37°C) and then with FITC/FP647-coupled beads and pH kinetic was followed for 2 h. Results indicated that MVs_MUC1_ significantly increased the phagosomal pH of X-DCs within the first 60 min (reaching 7.05 pH at 20 min) as compared to unpulsed X-DCs (*p* < 0.05), then decreasing and reaching the same values of unpulsed X-DCs at the end of chase. Uptake of soluble rMUC1 glycoprotein did not modify the acidic phagosomal microenvironment of X-iDCs (Figure 3A). To investigate whether the phagosomal pH increase observed in clinical grade X-DCs after MVs up-take was accompanied also by modulation of ROS molecules, MVs-uptake effects were studied in X-DC pretreated with DPI as shown in Figure 3B. DPI treatment of X-DCs significantly decreased phagosomal pH of X-DCs during the chase (*p* < 0.05). When DPI treated X-DCs were pulsed with MVs_MUC1_ ([X-DCs + DPI] + MVs_MUC1_) the phagosomal pH significantly increased in the first 60 min of chase (*p* < 0.05), although remaining lower than untreated X-DCs. These results indicated that up-take of MVs_MUC1_ also modulated antigen processing machinery of X-DCs by inducing alkalinization of the phagosomal microenvironment. The antigenic transfer of MUC1 was also investigated evaluating the percentage of MUC1 positive DCs following incubation with both MVs_MUC1_ and both rMUC1 by immunofluorescence studies, at 2 h and 12 h of incubation at 37°C (Figure 3C).

At 2 h pulsing, the percentage of X-DCs that had internalized MVs_MUC1_ was lower than the corresponding S-DCs (*p* < 0.05). A similar trend in decrease was also observed when the soluble rMUC1 was employed as immunogen. At 12 h of pulsing this difference was enhanced: the percentage of MUC1 positive X-DCs was much lower than MUC1 positive S-DCs for both MVs_MUC1_ and rMUC1 glycoprotein (*p* < 0.01 and *p* < 0.005, respectively). Interestingly, MUC1 antigenic transfer to X-DCs appeared to be more efficient when mediated by MVs_MUC1_ than the rMUC1 at 12h (*p* < 0.01). These results suggest that MVs_MUC1_ may be more efficient in antigenic transfer than the soluble rMUC1 glycoprotein, despite the fact that the intracellular availability of the MUC1 antigen is strongly reduced in X-DCs as compared to S-DCs.

### Tumor-derived MVs mediate MUC1 antigen cross-processing in clinical grade DCs and activation of MUC1 specific CD8^+^ T cells

Clinical grade DCs seems to show some “macrophages-like” features such as acid phagosomal pH and high ROS content in their phagosomal compartment (data not shown). We wanted to investigate further if this could affect their ability to cross-process tumor associated antigens (TAA). Both X-DCs and S-DCs were pulsed with MVs_MUC1_ and the rMUC1 soluble glycoprotein and intracellular distribution of the MUC1 antigen was observed by immunofluorescence, after 12 h.

**Figure 4:**
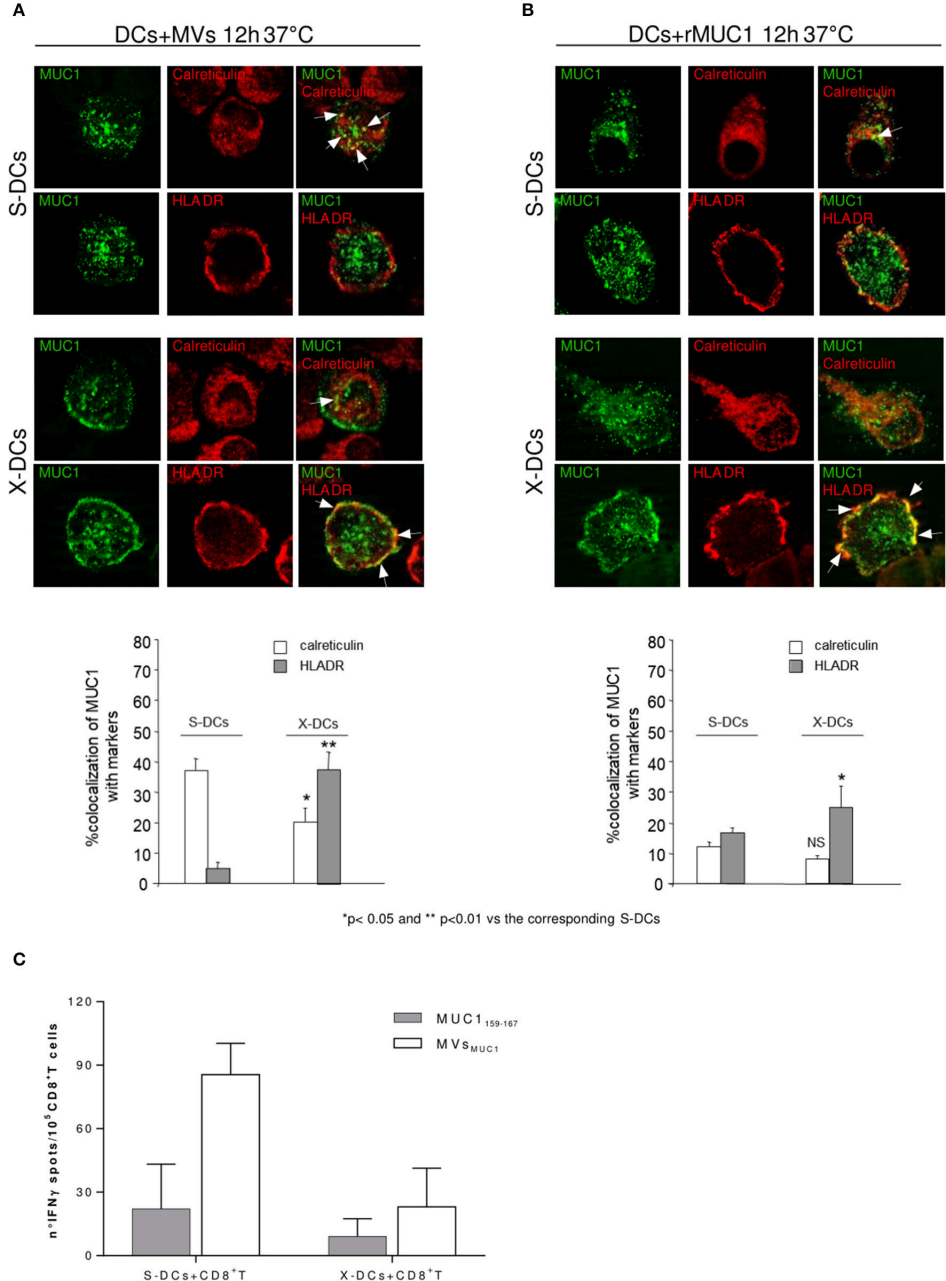
Clinical Grade DCs maintain the ability to cross-process antigen, when MUC1 is carried by MVs and to activate MUC1 specific CD8^+^ T cells. Intracellular localization of MUC1 carried by MVs_MUC1_ **(A)** or as soluble rMUC1 glycoform **(B)** in S-DCs (first and second row) and in X-DCs (third and fourth row) were visualized by immunofluorescence staining after 12 h of internalization employing the anti-MUC1 MoAb Ma552 (green) combined with antibodies specific for distinct intracellular compartment markers (red). In particular: anti-calreticulin polyclonal rabbit antibody, [calreticulin ER resident protein, employed as marker for HLA class I compartment] (first and third rows) and anti-HLAII-DR for HLA-II compartment (second and fourth rows). The percentage of colocalization (yellow) was calculated analyzing a minimum of 30 cells for each treatment randomly taken from three independent experiments. Results are expressed as mean values ± SE in histograms. Magnification, x63; Bar, 10 mm. **p < 0.01 and *p < 0.05 for X-DCs vs. the corresponding S-DCs pulsed with MVs_MUC1_ or rMUC1. **(C)** ELISpot assay to evaluate the IFNγ production by enriched MUC1-specific CD8^+^ T cells obtained from an ovarian cancer patient in response to mature S-DCs (left) or X-DCs (right) loaded with MUC1_159−167_ peptide (white histogram) or pulsed with MVs_MUC1_ (grey histogram). The average values of the experimental samples [(DCs + MUC1_159−167_) + CD8^+^T cells] and [(DC + MVs_MUC1_) + CD8^+^T cells] were subtracted of the corresponding background samples i.e., [unpulsed DCs + CD8^+^T cells] and [(DCs + MVs_DG75_) + CD8^+^T cells], respectively. Results were expressed as mean values ± SD of duplicates.

In S-DCs pulsed with MVs_MUC1_ (Figure 4A, row 1 and 2), MUC1 colocalized with calreticulin, marker of HLA class I compartment (38%) (Figure 4A, row 1), while scarce colocalization with HLA-DR, marker of HLA class II compartment (5%) was found (Figure 4A, row 2). When rMUC1 was employed to pulse S-DCs, low colocalization for both calreticulin and HLAII-DR compartment markers was found (<18%) (Figure 4B, row 1 and 2). These results confirmed previous observations indicating that only MUC1 supplied to DCs as cargo of MVs were routed to calreticulin^+^ compartment (27).

In X-DCs, following up-take of both immunogens, MUC1 colocalization was increased HLAII-DR positive compartment as compared to S-DCs (*p* < 0.01 for MVs_MUC1_ and *p* < 0.05 for rMUC1). In particular, MUC1 colocalized with HLAII-DR molecules in dots, close to the plasma membrane in X-DCs pulsed with MVs_MUC1_ (Figure 4A, row 4) as well as rMUC1 (Figure 4B, row 4). Interestingly, in X-DCs, colocalization of MUC1 with calreticulin positive compartment was observed only when X-DCs were pulsed with MVs_MUC1_, although at a lower extent of the corresponding S-DCs (*p* < 0.05). rMUC1 did not appear to colocalize significantly with calreticulin marker in X-DCs.

These results showed that while the soluble rMUC1 was mainly found in association with HLAII-DR, MVs_MUC1_ could be up-taken and cross-processed in HLA class I and II compartments by clinical grade X-DCs, although this was reduced compared to S-DCs.

To investigate whether the reduced cross-processing of MVs_MUC1_ in X-DCs was still sufficient to activate MUC1 specific T cell responses, CD8^+^ T cells were isolated by immunomagnetic selection from PBMCs of an ovarian cancer patient, previously vaccinated with the HLAI-A2 restricted MUC1_159−167_ peptide (31). MUC1 specific CD8^+^ T cells were expanded *in vitro* by a two round stimulation with autologous PBMCs pulsed with the MUC1_159−167_ peptide.

At the end of the culture, T cell activation was evaluated as IFNγ release in ELISpot assay (Figure 4C). The MUC1 enriched CD8^+^ T cells were stimulated by autologous X-DCs or S-DCs, loaded with immunogenic MUC1_159−167_ peptide or pulsed with MVs_MUC1._ T cells stimulated by unpulsed DCs or DCs pulsed with MVs_DG75_ (MVs from untransfected DG75 cells) were employed as background controls (for MUC1 peptide loaded and MVs_MUC1_ pulsed DCs, respectively). As shown in Figure 4C, X-DCs were less efficient as stimulator of IFNγ T-cell mediated response, independently by the MUC1 immunogen employed. MVs_MUC1_ appeared to perform better as immunogen than the exogenous MUC1_159−167_ peptide. In particular in [X-DCs+ MVs_MUC1_] induced a similar T response to [S-DCs + MUC1_159−167_], suggesting that the MUC1 carried by MVs could be processed and cross-presented to T cells also by X-DCs, whose processing and presentation abilities were dampened by culture conditions.

## Discussion

One of the critical key point in designing DCs-based antitumor vaccines is the choice of antigen formulation: the ideal immunogen should deliver a broad repertoire of TAAs combined to activatory signals in order to potentiate immunostimulatory capability of DCs. This strategy would reduce the possibility of immune escape and overcome HLA haplotype restriction that is a real limit for the peptide based DCs approach (33).

There is a compelling need to search for optimal immunogen formulations to efficaciously target and load DCs with antigens, and at the same time to activate them for improving anti-tumor DCs performance. Nowadays, that immune checkpoint blockade allows to clinically reverse the immune exhaustion, DC-based vaccines are being reassessed as a powerful approach to activate/maintain the unleashed antitumor memory T cell responses in order to control tumor disease progression (9, 34).

Cell released MVs display biological characteristics that make them as optimal candidate as immunogen platform able to simultaneously deliver multiple tumor antigens and immunostimulatory signals to DCs (35). Tumor-derived MVs can enhance the immunogenicity of soluble antigen (21, 22) and induce CD8^+^ T-cell responses in *in vitro* human studies toward tumor antigens (36). Delivery of the tumor antigens by MVs also modulate cross-presentation of those tumor glycosylated antigens such as MUC1 that are thought to induce only a tolerogenic CD4^+^ T cell response, although being relevant for tumor targeting (27). Furthermore, tumor-derived MVs have been shown to activate DCs *in vivo* by delivering tumor DNA triggering intracellular signaling cGAS/STING pathway resulting in potent anti-tumor responses (24–26).

In this study, we provide evidences that clinical grade culture conditions hamper DC immunogenicity by reducing phagocytosis and inducing a macrophage like feature of the phagosomal compartment i.e., strong acidification, besides altering DCs phenotype. We showed that tumor-derived MVs carrying the MUC1 tumor glycoantigen employed as immunogen, restored phagosomal pH close to neutrality, allowing cross-presentation of the tumor associated MUC1 glycoantigen and the activation of MUC1 specific CD8^+^ T cell response.

Culture conditions are critical for DC differentiation process from progenitor cells. It has described that *ex-vivo* DCs for clinical use are less immunogenic, because of a reduced expression of HLA and costimulatory molecules. Addition of human serum (autologous or pooled) to implement clinical grade DC performance appears to hamper cytokine production and reduce migratory capacity of DCs (37, 38). Indeed, the high plasticity of DCs, that enable them to quickly sense *in vivo stimuli*, can become a critical point in formulating experimental protocols for *ex-vivo* DC cultures (8). DCs generated in X-VIVO 15 serum free medium (X-DCs) displayed a spindle-like morphology and a distinct ability to respond to maturative pro-inflammatory cytokines than S-DCs (DCs grown in RPMI+10%FBS). Previous work had shown that DCs culture in X-VIVO 15 performed poorly in phenotype and cytokine secretion (10). Despite optimization of the culture protocol (anticipating cytokine re-addition during the culture; Napoletano C, unpublished), X-DCs still performed differently compare to S-DCs. Immature X-DCs had increased expression of the maturative CCR7 marker, while following maturation, CD14 marker was still maintained (*p* < 0.05). Also mature X-DCs showed a lower upregulation of costimulatory molecules than S-DCs.

This phenotype is associated to a reduced phagocytosis of immature X-DCs that usually is a functional feature of the mature DCs. This aspect can be quite relevant for the up-take of immunogens that are based on protein or particulated-based antigens. Most interestingly, the phagosomal machinery appeared to be modified by clinical grade culture conditions. X-DCs displayed a significant acidification of the phagosomal compartment that was maintained during the time following 3 μm beads phagocytosis, while phagosomal pH of S-DCs was close to neutrality and increased during the incubation time following 3 μm bead internalization.

Phagosome is a crucial compartment for the ability of DCs to cross-present antigens: it is considered a central hub for the cell where molecular cargos are docked and then sorted to other intracellular compartments of the cell (39). The cross-processing ability of DCs is finely tuned by a mild alkalinization of the phagosomal compartment. Induction of CD8^+^ T cells was obtained only by priming with monocyte-derived DCs with alkaline phagosomal pH, while macrophages, with an acid phagosomal pH, did not cross-process antigen (40). In mouse models, CD8^+^ DCs with a higher cross-processing ability showed an alkaline phagosomal pH (41). It has been hypothesized that alkaline pH delays protein degradation thus increasing the antigen amount available for cytoplasmic transportation and HLA-I association (39, 42, 43).

The results obtained suggest that X-DCs possess phagosomal machinery with strong similarities to macrophages, that quickly degrades the antigen thus favoring HLAII presentation and the induction of CD4^+^ T cells. Thus, the reduced ability to internalize the antigen combined to the increased efficiency in antigen degradation would imply a reduced immunogenicity of the DC vaccine designed. To investigate if this was the case, we employed two MUC1 based immunogens forms: a soluble rMUC1 glycoprotein, produced in CHO-K1 cells and MVs_MUC1_, tumor-derived MVs carrying the MUC1 antigen purified from a MUC1 stable transfected cell line. Size characterization by NTA indicated that vesicles were heterogenous and biochemical characterization of cell markers (data not shown) indicated that the prevalence of MVs_MUC1_ derived from plasma membrane exocytic pathways. Indeed, after 12 h pulsing, X-DCs showed a striking significant reduction of intracellular MUC1 distribution than S-DCs, for both the immunogens employed (MVs_MUC1_: *p* < 0.01; rMUC1: *p* < 0.005), strongly suggesting that the reduced up-take and the acidic compartment of X-DCs hasten degradation of MUC1 antigen.

We have recently shown that uptake of tumor-derived MVs exerts an immunostimulatory effect on antigen presentation by DCs, inducing a faster alkalinization of phagosomal compartment thus allowing cross-presentation of the MUC1 tumor glycosylated antigen (23). This mechanism could be of great relevance for shaping the immunogenicity of glycosylated tumor antigens.

We then asked whether, tumor-derived MVs could be a suitable immunogen formulation to counteract the phagosomal alkalinization and restore a pH value close to neutrality.

Uptake of MVs_MUC1_ by X-DCs significantly restored the phagosomal pH of X-DCs to neutrality in the first 60 min chase. This metabolic event is also supported by the observation that following pulsing of X-DCs with MVs_MUC1_, intracellular MUC1 distribution is significantly higher (*p* < 0.01) than X-DCs pulsed with rMUC1, thus suggesting that protein degradation has been lessened. Phagosomal pH is strictly dependent by Radical Oxigen Species (ROS) level produced in the phagosome by the combined and dynamically regulated function of NADPH oxidase 2 (NOX2) and VATPase (41, 44).

Indeed, MVs_MUC1_ uptake contributes to phagosomal ROS increase, as shown by NOX2 blocking experiments with the DPI inhibitor. However, other metabolic pathways triggered by MV uptake could be involved in the fine tuning of phagosomal ROS balance and pH regulation.

Most important, MVs_MUC1_ internalization allowed MUC1 cross-processing by X-DCs, despite these cells displayed a “macrophage-like” phagosomal compartment. After 12 h from MV internalization, in X-DCs MUC1 colocalized with HLA class II compartment (39%), but also with the calreticulin marker employed as HLA class I compartment (21%). In S-DCs MUC1 colocalization was prevalent with the calreticulin^+^ compartment (38%), as expected. The rMUC1 soluble glycoprotein was sorted exclusively in HLAII compartment both in S-DCs and in X-DCs. In DCs, cross-processing of soluble antigens occurs by distinct mechanisms: the “cytosolic” and “vacuolar” pathways. In the former, the internalized antigen sorted into the phagosome, then translocates in the cytosol where proteasome degradation occurs and proteolitic peptides are loaded by TAP dependent mechanism in the ER where the association to MHCI occurs. In the vacuolar pathway, exogenous antigens are degraded in the endosome, loading endosome resident MHCI molecules (39, 45). The co-localization of MUC1 with the ER marker calreticulin, and the detection of MUC1 in the cytosolic fraction of MVs_MUC1_ pulsed DCs previously shown (27), clearly suggested that the cytosolic pathway was involved in the MUC1 cross-processing mediated by MVs delivery, although the vacuolar pathway could not be excluded. These intracellular events resulted in cross-presentation of the MUC1 antigen since X-DCs pulsed with MVs_MUC1_ were able to activate CD8^+^ T cells specific for the HLA-A2 restricted MUC1_159−167_ epitope, although with a lower efficiency than S-DCs, as expected.

Thus, delivery of antigenic cargo through MVs appeared to be a possible strategy to empower antigen presenting ability of DCs for clinical use.

Tumor-derived MVs immunogenicity could be possibly enhanced by mean of genetic and biochemical interventions with the ultimate goal to generate an off the shelf/cell free immunogen (46, 47). Induced genome instability of tumor cells could increase the amount of novel neoantigens that elicit strong immune response (48), thus increasing the antigenic cargo of the released MVs. Also, modulation of glycosylation is an appealing option to harness MVs immunogenicity (49). So far, glycosylation is regarded as a complex and finely tuned signaling code among cells and microenvironment, not just a “default cell décor” (50). DCs are endowed of specific receptor, C-type lectin, recognizing selectively the distinct glycan moieties (51). By specific and selective ligand receptor interaction, glycan *repertoire* shapes immunogenicity of the antigens by modulating their internalization and at the same time triggering activatory/inhibitory signals to the DCs (52–54). Selective genome editing strategies allow to control glycan synthesis, thus obtaining cells (and therefore MVs) with the desired glycan profile and defined immunoregulatory properties (55).

In summary, we have investigated the immunomodulatory impact of tumor-derived MVs carrying MUC1 as immunogen in clinical grade culture condition DCs. Results indicate that optimization of the MUC1 antigen cross-processing could be induced upon tumor derived MVs_MUC1_ internalization in clinical grade X-DCs, despite their acidic phagosomal compartment, that is a feature of macrophage cells. This effect appears to be dependent by metabolic changes triggered by phagosomal ROS increase and alkalinization. Furthermore, MVs_MUC1_ pulsed DCs could stimulate MUC1 specific CD8^+^ T cells to produce IFNγ response. We believe these results to further support the exploitation of tumor-derived MVs as optimal immunogens for DC-based anti-cancer vaccine.

## Author contributions

All authors contributed with their specific expertise to study design, data collection, analysis, and interpretation of results and to critically evaluate and approve the manuscript prior publication. AR designed and supervised the study and wrote the manuscript. FeB and CN developed the methodology for phagosomal pH and ROS detection and performed DC studies. MD and CDA were responsible for cell and DC culture, phagosomal pH measurments, ROS detection in DC phagosome, flow cytometry analysis. HRK, IGZ, and IR performed microvesicles production, isolation and biochemical characterization. ADF performed flow cytometry analysis of cell and microvesicles. MRT and FrB were responsible for the immunofluorescence studies. CA performed MUC1^+^ CD8^+^ T cell enrichment and IFNγ ELISpot, together to HRK. PBP provided cancer patient blood samples and clinical information. MN provided valuable support for the study design and interpretation of results.

### Conflict of interest statement

The authors declare that the research was conducted in the absence of any commercial or financial relationships that could be construed as a potential conflict of interest.
